# High-Sensitivity Flow Cytometry for the Reliable Detection of Measurable Residual Disease in Hematological Malignancies in Clinical Laboratories

**DOI:** 10.3390/diseases12120338

**Published:** 2024-12-22

**Authors:** María Beatriz Álvarez Flores, María Sopeña Corvinos, Raquel Guillén Santos, Fernando Cava Valenciano

**Affiliations:** 1 Centro Nacional de Investigaciones Cardiovasculares Carlos III, 28029 Madrid, Spain; 2 URSalud Laboratory, Hospital Universitario Infanta Sofia, 28702 Madrid, Spain; msopena@ursalud.com (M.S.C.); rguillen@salud.madrid.org (R.G.S.); ferjulte@gmail.com (F.C.V.)

**Keywords:** measurable residual disease, hematology, leukemia, lymphoma, flow cytometry, limit of detection, bone marrow, multiple myeloma, mastocytosis, staining and labeling, high-sensitivity flow cytometry

## Abstract

Background: Monitoring of measurable residual disease (MRD) requires highly sensitive flow cytometry protocols to provide an accurate prediction of shorter progression-free survival. High assay sensitivity generally requires rapid processing to avoid cell loss from small bone marrow sample volumes, but this requirement conflicts with the need in most clinical cytometry laboratories for long processing and acquisition times, especially when multiple MRD studies coincide on the same day. Methods: The proposed protocol was applied to 226 human bone marrow and 45 peripheral blood samples submitted for the study of MRD or the detection of rare cells. Samples were processed within 24 h of extraction and acquired with an eight-color flow cytometer. Results: The FACSLyse-Bulk protocol allows for the labelling of millions of cells in under 90 min in small sample volumes without affecting the FSC/SSC pattern or antigen expression, and it also allows antigens to be fixed to the membrane, thus avoiding the capping phenomenon. Conclusions: The proposed protocol would allow clinical flow cytometry laboratories to perform MRD studies in house and easily achieve a limit of detection and limit of quantification <0.001%, thus avoiding the need to outsource analysis to specialized cytometry laboratories.

## 1. Introduction

Clinical laboratories face increasing demands for greater sensitivity in the detection of small numbers of residual malignant cells—known as measurable residual disease (MRD)—after treatment for leukemia or lymphoma. The sensitivity of MRD detection has increased with the introduction of digital cytometers that allow for the analysis of eight or more colors and marking protocols that permit the analysis of millions of cells [1,2].

The application of bulk lysis protocols is crucial for the accurate study of MRD in leukemia by flow cytometry, especially when a high number of cells must be acquired to ensure sensitivity. Bulk lysis efficiently removes erythrocytes and non-nucleated cells, ensuring that the analysis focuses solely on nucleated cells, which is essential for detecting rare leukemic cells present at very low frequencies.

Acquiring a large number of cells significantly enhances the sensitivity of MRD detection as it increases the likelihood of identifying rare leukemic populations that might otherwise be missed. An insufficient number of cells reduces the reliability of MRD detection, increasing the risk of false negatives.

Bulk lysis also facilitates the processing of large sample volumes, allowing for the acquisition of millions of cells. This is critical not only for the precise quantification of MRD but also for the robust assessment of leukemic subpopulations. In a clinical setting, this approach maximizes the accuracy of MRD detection while ensuring efficient, large-scale cell acquisition, which is necessary for informed therapeutic decision making. Highly sensitive methodologies used to detect MRD in multiple myeloma patients include next-generation flow cytometry (NGF) and next-generation sequencing (NGS), which have enabled the detection of MRD with sensitivity thresholds of up to 10^−5^ and 10^−6^ [1,2]. The use of these methodologies has improved progression-free survival (PFS) and overall survival in patients attaining MRD negativity, leading to the broader adoption of MRD assessment. Together, these studies highlight the clinical value of MRD assessment as a critical prognostic indicator for patient survival [3].

However, the long staining and acquisition times required for flow cytometry analysis and the high service demand mean that many hospital laboratories are unable to carry out routine MRD studies and have to outsource analysis to specialist laboratories. The well-established EuroFlow-NGF protocol requires more than 3 h for complete sample processing [2]. The limit of detection (LOD) is the lowest signal that can be detected above the background. Consensus recommendations stipulate an LOD in MRD studies of <0.001%, which requires the acquisition of at least 3 × 10^6^ bone marrow cells. This in turn implies that flow cytometry approaches for detecting MRD in multiple myeloma should ideally have a limit of quantification (LOQ, the lowest signal that can be precisely and accurately measured) of 0.001%, requiring the acquisition of a minimum of 5 × 10^6^ bone marrow cells [4,5].

The aim of this study was to establish a straightforward protocol to allow clinical flow cytometry laboratories, equipped with standard resources (a flow cytometer with at least eight color channels) and limited personnel, to conduct MRD analyses. In this report, we present FACSLyse-Bulk, a protocol for the rapid staining of large numbers of cells in bone marrow and peripheral blood samples that can be adapted to the equipment and schedules of routine clinical diagnostic laboratory practice.

## 2. Results

### 2.1. Staining Quality and Cell Number

The FACSLyse-Bulk protocol permits the sample preparation of large numbers of cells for multiparametric flow cytometry, including intracytoplasmic antibody labelling, in under 90 min. In all samples studied, correct staining was achieved with the supplier-recommended antibody amounts for just 1 × 10^6^ cells. Although the lysing solution contains formaldehyde, the markers studied showed no evidence of tandem breaks. Membrane fixing by the lysing solution prevents capping and receptor-mediated endocytosis and also allows sample acquisition to be delayed until the cytometer is available. The protocol generates less debris and fewer doublets than described in the Bulk Lysis Euroflow protocol [2] (Table 1 and Figure S1) and does not significantly affect cell complexity or labelling resolution (Figure S2).

**Figure 1:**
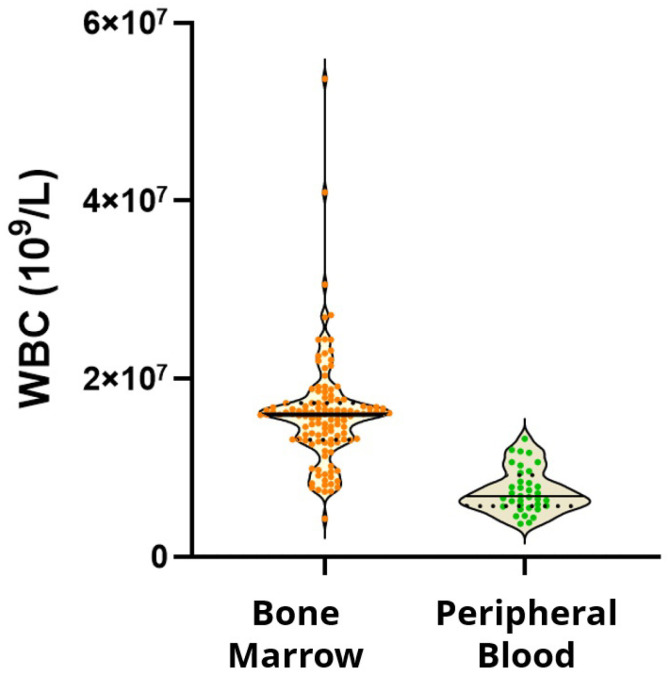
Violin plot representation of stained white blood cells detected with the FACSLyse-Bulk protocol in bone marrow and peripheral blood.

The white blood cell count stained with this protocol in bone marrow was 15,961,484 ± 6,051,776, and that in peripheral blood was 7,418,500 ± 2,475,834 (mean and standard deviation); the minimum-to-maximum range in each case was 4,260,000–53,712,000 and 3,710,000–13,260,000. As these were real clinical samples and we did not want to risk losing any sample due to a staining error, no maximum staining limit was enforced. It is possible that the protocol may allow for staining of a significantly higher number of cells (Figure 1).

The results from a bone marrow sample containing 13 million cells (excluding debris and doublets) are shown in Figure S2. The analysis shows that the cell populations can be resolved by reducing the number of events shown, greatly facilitating their assessment. The figure also illustrates how the protocol preserves the sample and ensures a clean staining, an important feature given that protocols that provoke more cell death are associated with cell clumping, non-specific staining (false positives), autofluorescence, and decreased antigen expression [6].

**Figure 2:**
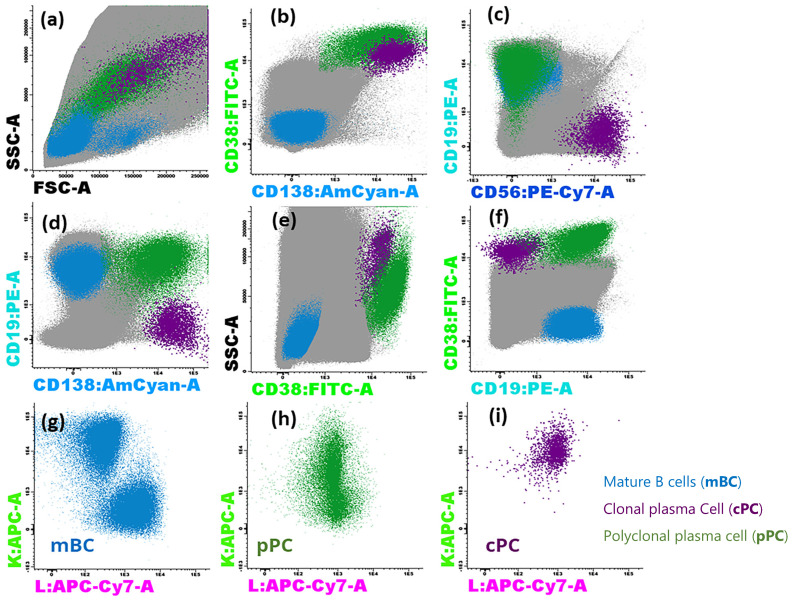
Example of FSC/SSC distribution (**a**) and phenotypic expression profiles of CD138, CD38, CD19, and CD56 (**a**–**f**) and intracellular light chains (**g**–**i**) in bone marrow from a multiple myeloma patient during the study of MRD with the FACSLyse-Bulk protocol. Polyclonal plasma cells are depicted in green, mature B lymphocytes in blue, and clonal plasma cells in dark purple. Clonal plasma cells constitute 1.84% (3001 events) of the total population (12.41 million events), excluding debris and doublets, and have an altered CD19−CD56+CD45−and low CD38 phenotype with a kappa light chain restriction.

The lack of any significant distorting effect of FACSLyse-Bulk on fluorescence is illustrated in Figure 2, which shows the appropriate identification of mature B-cells, clonal plasma cells, and polyclonal plasma cells in a sample of more than 12 million acquired events.

**Figure 3:**
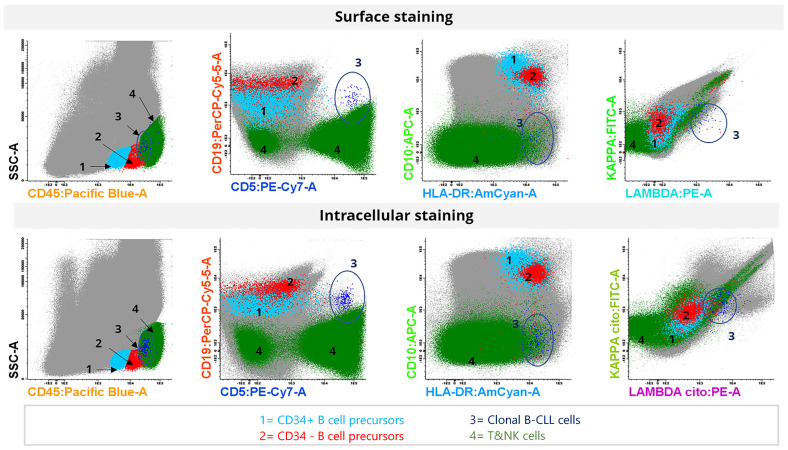
Comparison of antigenic expression intensity between surface staining (**top**) and intracellular staining (**bottom**) of MRD in human bone marrow from a patient with B-cell chronic lymphocytic leukemia. The sample contained no mature B lymphocytes; however, CD34+ (zone 1, cyan) and CD34- (zone 2, red) B-cell precursors were detected. A limited number of residual clonal cells from B-cell chronic lymphocytic leukemia (CD19+CD5+CD10−CD45+) were observed post-treatment (zone 3, blue and circle). The remaining lymphocytes, including T and NK cells, are represented in zone 4 (green). Both surface and intracellular staining accurately identify the clonal cells, and fluorescence intensity is comparable across all antigens studied in the different subpopulations.

We also found no marked differences between antigenic expression intensities detected by surface or intracellular staining (Figure 3). Similarly, the percentage and number of events in the different populations analyzed did not vary significantly between surface and intracellular staining (Table S1).

### 2.2. Limit of Detection and Limit of Quantification

**Figure 4:**
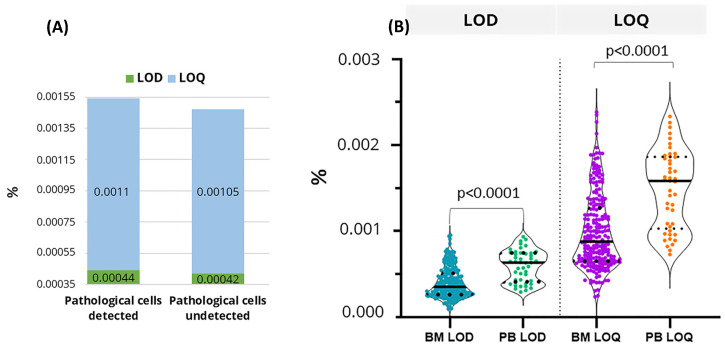
(**A**) In samples with no detected pathological cells, more events were acquired, resulting in lower LOD and LOQ than in samples in which more than 50 clonal events were detected. (**B**) Violin plot representation of LOD and LOQ in bone marrow and peripheral blood. *p* < 0.0001 (Mann–Whitney U test). LOD: Limit of detection; LOQ: limit of quantification; BM: bone marrow; PB: peripheral blood.

For the LOD, several studies cite a minimum of 20 events for the identification of a homogeneous, clustered population of cells reliably detectable by flow cytometry [7,8,9], whereas the LOQ can be obtained from a cluster of 50 events if a sufficient total number of events is acquired [4,8,10,11]. With the FACSLyse-Bulk protocol, an LOD < 0.001 was easily achieved in 99.26% of the samples studied. In some cases, there was no need to acquire a large number of events because pathological cells were abundant, whereas the larger number of acquired events in samples with no detected pathological cells resulted in lower LOD and LOQ values (Figure 4A). Of the 271 patient cases studies, no pathological cells were detected in 103 of them.

In the 226 bone marrow and 45 peripheral blood samples tested, the mean LOD values were 0.0003978% and 0.0009946%, respectively, and the mean LOQ values were 0.0005957% and 0.0014891% (Table 2 and Table S2 for more detail). Both the LOD and the LOQ were lower in studies conducted in bone marrow than in those conducted in peripheral blood (Figure 4B). This difference is due to the higher proportion of red blood cells in peripheral blood, which requires a larger sample volume to achieve comparable sensitivity. The present study primarily focused on the analysis of bone marrow; however, LOD and LOQ values in peripheral blood could be improved by increasing the sample volume. This approach is feasible as peripheral blood is generally more readily available, providing an advantage for studies that require substantial sample quantities.

**Figure 5:**
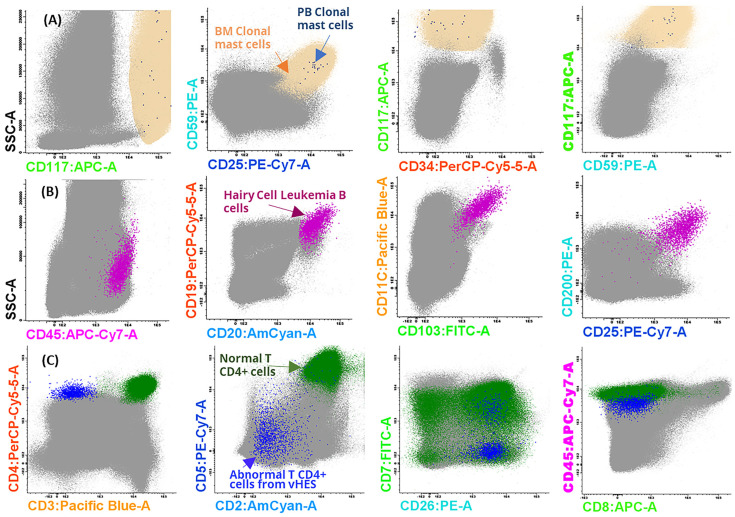
**Example of rare cell identification in peripheral blood and bone marrow samples with the FACSLyse-Bulk protocol.** (**A**) Detection of mast cells with an aberrant phenotype (blue dots) in the peripheral blood of a patient with systemic mastocytosis (0.0007%; 20 events from 3,073,318 total cells in a 1200 µL sample volume). The phenotype is compatible with that of mast cells detected in the bone marrow (reference image in cream). (**B**) Study of peripheral blood from a patient with hairy cell leukemia (magenta) after treatment (0.0428%; 2149 events from 4,772,959 total cells in an 800 µL sample volume). (**C**) Abnormal T CD4+ lymphocytes (blue) in bone marrow from a patient with lymphocytic-variant hypereosinophilic syndrome (vSHE) (0.00960%; 1492 events from 15,229,628 total cells in a 300 µL sample volume). Normal T CD4+ lymphocytes are depicted in green. BM: Bone marrow; PB: peripheral blood.

The volume of most bone marrow aspirates was below 2 mL. In most cases, this did not present a limitation because the mean sample volume needed for staining 8 million cells was 695.38 µL (Table 2). A larger volume (1347.22 µL) was required for the peripheral blood samples because cell counts are usually lower in blood than in bone marrow. Even with these low sample volumes, FACSLyse-Bulk was able to identify clonal mast cells and villous lymphocyte cells in peripheral blood and even clonal T-lymphocytes of lymphocytic-variant hypereosinophilic syndrome in bone marrow samples (Figure 5).

Due to the time restrictions in the diagnostic hematology laboratory, the whole tube could be acquired for only a few samples, resulting in lower LOD and LOQ values than would have been obtained had the entire tube been acquired. Advantages of this protocol include the ability to handle smaller volumes of both the lysing solution and wash buffer (using 5 mL tubes instead of 50 mL), which simplifies and speeds up the process and reduces potential sample handling errors. In addition, only one type of lysing solution is required and, because there is no need to adjust the final cell concentration for labelling, the sample can be stained directly.

### 2.3. Protocol Limitations

As limitations to consider in this protocol, it does not specify a maximum limit for the number of cells that can be stained. Additionally, it is advisable to test fluorochromes other than those included in the present study, as only the most commonly tested and validated fluorochromes for clinical cytometry have been used.

## 3. Discussion

There is a critical need to enhance the sensitivity of flow cytometry for the detection of MRD and underrepresented clonal cell populations in order to ensure the precise monitoring of treatment responses and hematological diagnosis. The FACSLyse-Bulk protocol was developed to meet the demand for the completion of MRD assessments in shorter time frames, enabling their performance by a standard clinical flow cytometry laboratory and ensuring same-day analysis, even when bone marrow specimens are received late during regular working hours. FACSLyse-Bulk allowed for accurate MRD reporting in multiple myeloma cases and demonstrated utility in identifying rare cell populations. Furthermore, its robustness has been validated through external quality assessments for MRD studies that our laboratory has participated in.

Diagnostic and MRD assessments of bone marrow require various assays (including morphological analysis, molecular profiling, and flow cytometry) that often require substantial sample volumes that may not always be achievable. Furthermore, efforts to obtain larger volumes can lead to hemodilution, which can yield false negative MRD results [12,13]. The ability to conduct these assessments with minimal bone marrow aspirate volumes would facilitate the completion of all essential tests without exclusions due to sample limitations while also reducing the risk of hemodilution. This approach would ultimately enhance the quality and reliability of sample analysis.

With the FACSLyse-Bulk protocol, antigen internalization is prevented, there is no observed tandem breakage, and the shorter sample processing time frees up laboratory time for sample acquisition, which can take more than an hour per tube (depending on the cell concentration and cytometer signal-processing speed). These features make FACSLyse-Bulk a highly sensitive protocol accessible to any clinical flow cytometry laboratory. This protocol generates minimal debris and doublets while maintaining FSC/SSC cell profiles and fluorescence quality.

The FACSLyse-Bulk protocol differs from the well-established EuroFlow Bulk Lysis protocol in several respects. FACSLyse-Bulk uses a single lysis solution (FACS lysing) instead of two (ammonium chloride and FACS lysing). The new protocol also uses a lower volume of lysis solution, just 2 mL per test, compared with 48 mL per test for the EuroFlow protocol. Sample handling is minimized by the elimination of the need to transfer cells from a 50 mL tube to a 5 mL tube. Additionally, in FACLyse-Bulk, there is no need to adjust the cell count for staining after cell lysis. These features significantly reduce the risk of handling errors, a critical consideration in protocols involving multiple steps.

MRD studies have a major impact on clinical decision making. In patients with acute myeloid leukemia (AML), the detection of MRD by multiparametric flow cytometry after the achievement of complete remission with induction chemotherapy is strongly associated with an increased cumulative incidence of relapse. Studies using leukemia-associated immunophenotypes and similar approaches confirm the adverse prognostic impact of pre-transplant MRD positivity [12]. It has also been demonstrated that MRD-negative patients have significantly higher 5-year disease-free survival and overall survival rates than MRD-positive patients, regardless of age, AML subtype, timing of MRD assessment, or method [14]. A retrospective multinational study demonstrated the prognostic utility of evaluating MRD in multiple myeloma, thereby helping to inform clinical decision making to enhance patient outcomes. Among patients monitored for MRD during frontline therapy, those achieving MRD negativity had notably better progression-free survival (PFS) than those with persistent MRD. Clinical decisions based on MRD status, such as treatment adjustments, were associated with improved PFS in patients in whom changes were implemented, as opposed to those in whom MRD findings were not acted upon. Furthermore, in MRD-positive patients undergoing maintenance therapy, treatment intensification or modification significantly enhanced PFS [15]

Regarding the future of MRD assessment by flow cytometry, several studies of B-acute lymphoblastic leukemia have demonstrated strong agreement between MRD results obtained from bone marrow and those from peripheral blood. These findings support the use of peripheral blood as a less invasive alternative for ongoing disease monitoring, which is especially advantageous for patient management and therapeutic decision making. By adopting this approach, clinicians can facilitate more frequent assessments while also avoiding the discomfort associated with repeated bone marrow aspirations [16,17].

The possible transition of MRD assessment from bone marrow to peripheral blood is thus likely to significantly increase sample availability and monitoring frequency, making the optimization of protocol durations an especially urgent goal. Implementing more efficient workflows can assist in managing this anticipated increase in peripheral blood assessments while simultaneously maintaining the quality of laboratory services.

## 4. Materials and Methods

### 4.1. Patients and Samples

A total of 271 human samples was submitted to the central laboratory of the Community of Madrid at Hospital Infanta Sofía (Madrid), which serves as the central repository for all specimens collected from the six public hospitals within the community. These samples comprised 226 heparin-anticoagulated bone marrow specimens and 45 peripheral blood EDTA samples, which were intended for the study of MRD and rare populations, such as those observed in mastocytosis (Table 3). All patient samples submitted for MRD studies were diagnosed previously according to the WHO criteria. All samples were processed according to the FACSLyse-Bulk protocol and acquired within 24 h of extraction.

### 4.2. FACSLyse-Bulk Protocol

The required volume was dispensed for each patient sample (with a recommended minimum of 8 million white blood cells). The recommended minimum cell count is 3 million, as this is required to achieve an LOD below 0.001 [3]. The sample volume depends on the cell concentration. In all cases, a cell count was performed with a hematological analyzer (Advia 2120I), and bone marrow samples were filtered by passing them through an insulin needle (Microlance 3; BD, San Jose, CA, USA). Cells were washed twice with wash buffer, consisting of 5 mL of PBS or BD FACS™ sheath fluid (BD Biosciences) supplemented with 3% bovine serum albumin and centrifuged for 5 min at 1800 rpm, followed by removal of the supernatant with a Pasteur pipette.

Cell surface antigens were labelled by the incubation of samples with appropriate fluorescence-conjugated antibodies for 15 min at room temperature (RT) in the dark (the antibody concentration in µL/test should be as recommended by the manufacturer for staining one million cells). The cells were then washed again with wash buffer and centrifuged for 5 min at 1800 rpm. After removing the supernatant, the cells were dislodged by gentle vortexing, and 2 mL of lysis buffer (FACS lysing solution (BD Biosciences) diluted 1:10 in distilled water) was added. After incubation for 6 min at RT, the cell suspension was centrifuged at 1800 rpm for 5 min, the supernatant was removed, lysis was stopped by the addition of 5 mL wash buffer, the permeabilized cells were centrifuged again in the same conditions, and the supernatant removed. After this, the cells were incubated for 15 min at RT with appropriate antibodies targeting intracytoplasmic antigens and washed again. At the end of the procedure, the cells were resuspended in 2 mL of wash buffer, and samples were analyzed by flow cytometry as described below. If the analysis only requires targeting of the surface antigens, the intracellular labelling and subsequent washing step can be omitted. The FACSLyse-Bulk staining scheme is summarized in Scheme S1.

### 4.3. Flow Cytometry

All patient samples were analyzed by eight-colour flow cytometry in a BD FACSCanto II™ flow cytometer (BD Biosciences, San Jose, CA, USA), and data were analyzed with Infinicyt™ software (Version 2.0.6.b) (BD Biosciences, San Jose, CA). In all samples, debris was excluded by gating in the FSC/SSC dot plot, and doublets were discriminated in an FSC-Area/FSC-High dot plot.

#### Cytometer Standardization

To generate comparable results from different patient samples analyzed at different times, we used the BD Cytometer Setup and Tracking System (CS&T), following the recommendations in BD FACSDiva™ Software Version 6. The photomultiplier tube (PMT) voltages were optimized to resolve dim cell populations using unlabeled lysed whole blood cells (Technical Bulletin Standardizing Application Setup Across Multiple Flow Cytometers Using BD FACSDiva™ Version 6 Software, BD Biosciences). The resulting MFI target values were used to maintain instrument standardization in subsequent calibrations [18,19]. The cytometer configuration and mean fluorescence intensity (MFI) target values are detailed in Table S3.

Cells were acquired at low speed to avoid the formation of aggregates and to reduce the number of electronic aborts. Given the long acquisition time needed to acquire millions of cells, the acquisition had to be stopped and the tube had to be shaken to prevent the cells from sedimenting and clogging the acquisition line. A pause of 6 s is recommended before data recording to allow time for the flow sample to stabilize. Sample flow was monitored with FSC vs. time plots (Figure S3).

### 4.4. Statistical Analysis

The statistical significance of differences was determined with the Mann–Whitney U test. Statistical differences were considered significant at *p* < 0.05. Statistical comparisons were performed with SPSS version 21.0 (IBM, Armonk, NY, USA), and some graphical representations were generated with GraphPad Prism version 10.2.3 (GraphPad, San Diego, CA, USA).

## 5. Conclusions

The FACSLyse-Bulk protocol addresses the critical need for increased sensitivity in flow cytometry for the detection of MRD and rare clonal cell populations in hematological disorders. By enabling accurate same-day analysis, even with late-received bone marrow samples, this protocol offers a practical solution that allows clinical laboratories to independently perform MRD analyses without the need for outsourcing. The advantages of FACSLyse-Bulk include the use of small sample volumes, the prevention of antigen internalization, and shorter processing times, all of which contribute to improved workflow efficiency and accessibility for routine use in clinical flow cytometry laboratories. The protocol’s reliability and robustness have been validated across various clinical applications, ensuring its utility for precise disease monitoring.

## Figures and Tables

**Table 1:** Distribution of percentage values for debris, cell doublets, and total events; total number of events (excluding doublets and debris); and sample volume in 51 bone marrow samples processed with the FACSLyse-Bulk protocol.

	%Debris	%Doublets	% Total Events	Total No. of Events	Sample Volume (µL/test)
Mean	4.84	1.17	93.99	8,097,651.92	634.60
Range	(1.05–33.43)	(0.27–2.76)	(65.50–97.88)	(4,189,186–21,212,792)	(140.00–1800.00)
N	51	51	51	51	50

**Table 2:** Descriptive statistics for sample volume, total acquired events, LOD, and LOQ in bone marrow and peripheral blood obtained with the FACSLyse-Bulk protocol.

	Bone Marrow				Peripheral Blood			
	Sample Volume Added (µL)	Total Events Acquired *	LOD (%)	LOQ (%)	Sample Volume Added (µL)	Total Events Acquired *	LOD (%)	LOQ (%)
Mean	695.38	6,072,596.57	0.0003978	0.0009946	1347.22	3,734,489.78	0.0005957	0.0014891
SD	387.45	2,882,620.15	0.0001749	0.0004372	341.00	1,306,281.78	0.0001827	0.0004569
Minimum	75.00	2,096,816.00	0.0009538	0.0023846	600.00	2,141,538.00	0.0009339	0.0023348
Maximum	2000.00	21,212,792.00	0.0000943	0.0002357	2000.00	6,859,463.00	0.0002916	0.0007289
N	131	226	224	224	36	45	45	45

* Excluding debris and doublets; **LOD**: limit of detection (20 events); **LOQ**: limit of quantification (50 events); **SD**: standard deviation.

**Table 3:** Distribution of samples analyzed with the FACSLyse-Bulk protocol according to disease and origin.

Disease	Sample		N
	Bone Marrow	Peripheral Blood	
Multiple Myeloma	131	0	131
Hairy Cell Leukemia	4	19	23
B-Cell Chronic Lymphocytic Leukemia	12	14	26
B-cell Non-Hodgkin Lymphoma	6	2	8
Acute Lymphoblastic Leukemia	25	0	25
Acute Myeloid Leukemia	13	0	13
Mastocytosis	2	1	3
Diffuse Large B-Cell Lymphoma	4	0	4
Lymphocyte-Variant Hypereosinophila	1	1	2
T-cell non-Hodgkin Lymphoma	1	0	1
Mantle Cell Lymphoma	3	2	5
Marginal Cell Lymphoma	7	3	10
Follicular Lymphoma	17	3	20
TOTAL	226	45	**271**

## Data Availability

The original contributions presented in the study are included in the article; further inquiries can be directed to the corresponding author.

## Supplementary Materials

The following supporting information can be downloaded at: https://www.mdpi.com/article/10.3390/diseases12120338/s1, Figure S1: The distribution of cell events, cell doublets and debris for the FACSLyse-Bulk protocol and Bulk Lysis Euroflow protocol; Figure S2: Example of preserved FSC/SSC and CD45 expression profiles after completion of the FACSLyse-Bulk protocol in a non-LNH-B infiltrated human bone marrow sample; Table S1: Comparison between percentage and number of events of different populations according to surface or intracellular staining from a sample of a patient with B-CLL.; Table S2: Detailed descriptive statistics of LOD and LOQ in bone marrow and peripheral blood samples.; Scheme S1: Annotated FACSLyse-Bulk protocol scheme.; Table S3: FACSCanto II Cytometer configuration and Mean Fluorescence Intensity target values.; Figure S3: FSC vs Time monitoring of fluid stability.
