# Supplementary material for: High-Sensitivity Flow Cytometry for the Reliable Detection of Measurable Residual Disease in Hematological Malignancies in Clinical Laboratories
Source: Diseases. 2024 Dec 22;12(12):338. doi: 10.3390/diseases12120338 (PMC11727296; doi:10.3390/diseases12120338)
Supplement: Supplementary file 1 [file diseases-12-00338-s001.zip › diseases-3238725-supplementary.pptx]

## Slide 1
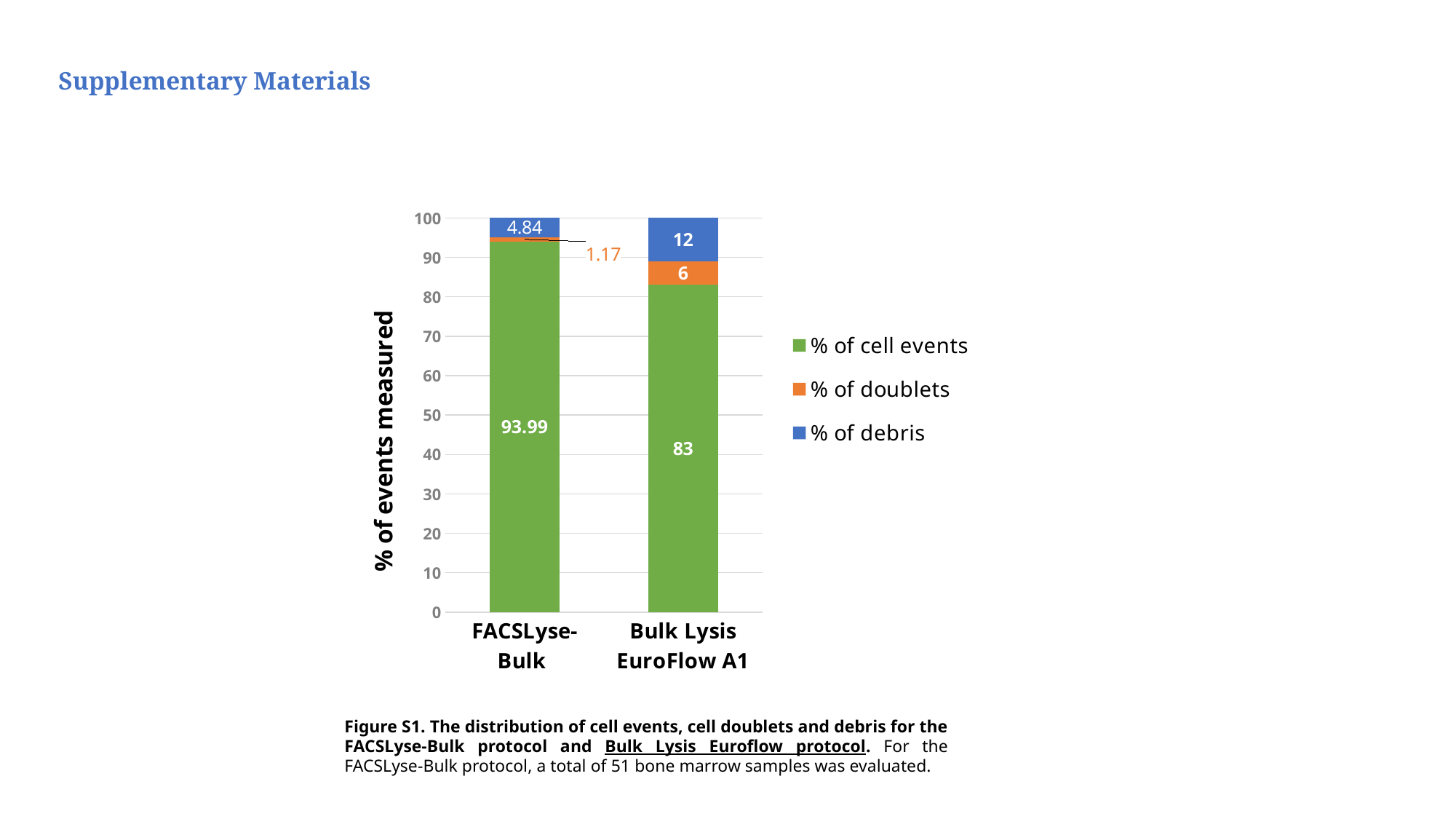

Supplementary Materials
### Chart
| Category | % of cell events | % of doublets | % of debris |
|---|---|---|---|
| FACSLyse-Bulk | 93.99 | 1.17 | 4.84 |
| Bulk Lysis EuroFlow A1 | 83.0 | 6.0 | 12.0 |Figure S1. The distribution of cell events, cell doublets and debris for the FACSLyse-Bulk protocol and Bulk Lysis Euroflow protocol. For the FACSLyse-Bulk protocol, a total of 51 bone marrow samples was evaluated.

## Slide 2
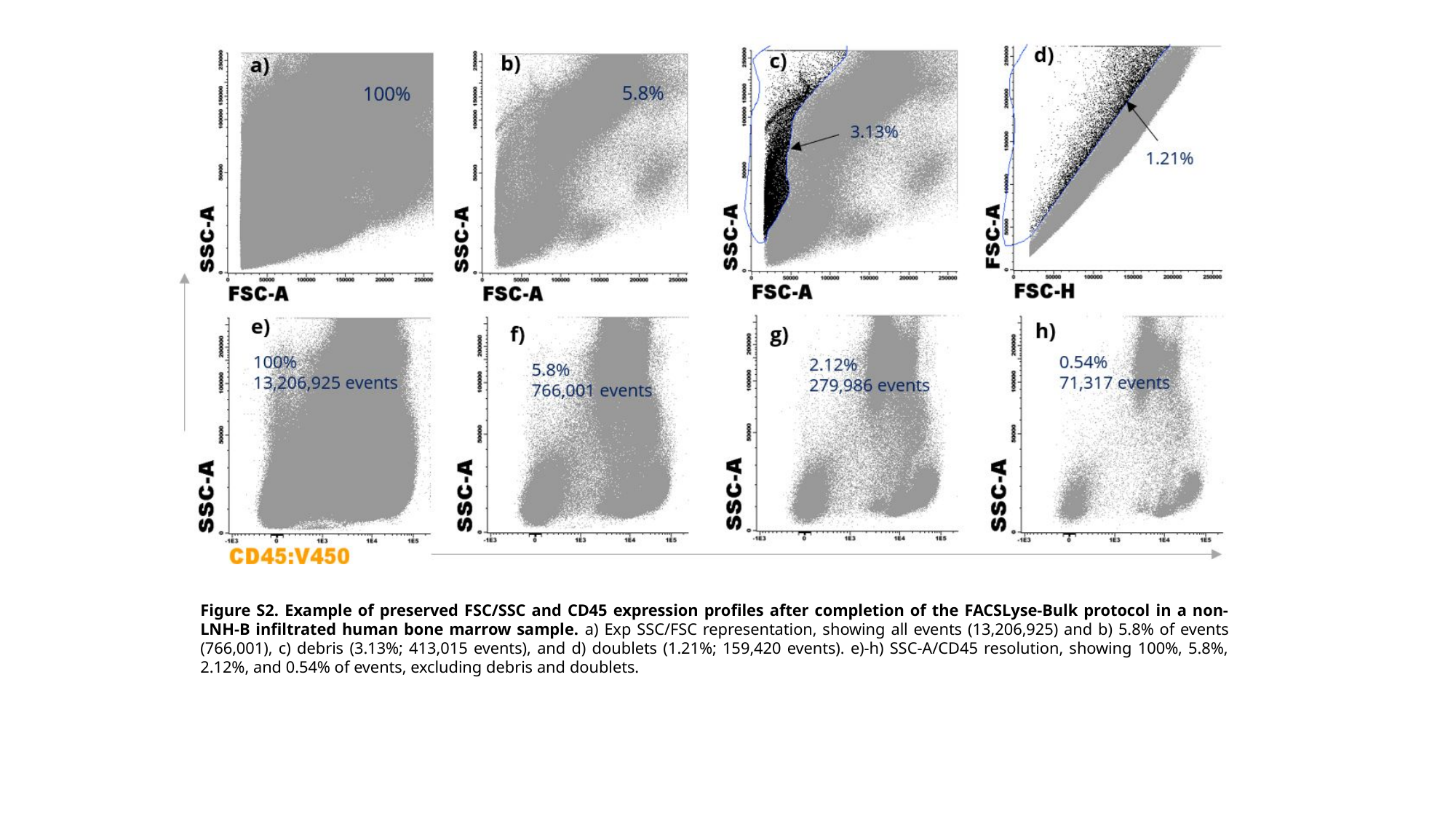

Figure S2. Example of preserved FSC/SSC and CD45 expression profiles after completion of the FACSLyse-Bulk protocol in a non-LNH-B infiltrated human bone marrow sample. a) Exp SSC/FSC representation, showing all events (13,206,925) and b) 5.8% of events (766,001), c) debris (3.13%; 413,015 events), and d) doublets (1.21%; 159,420 events). e)-h) SSC-A/CD45 resolution, showing 100%, 5.8%, 2.12%, and 0.54% of events, excluding debris and doublets.

## Slide 3
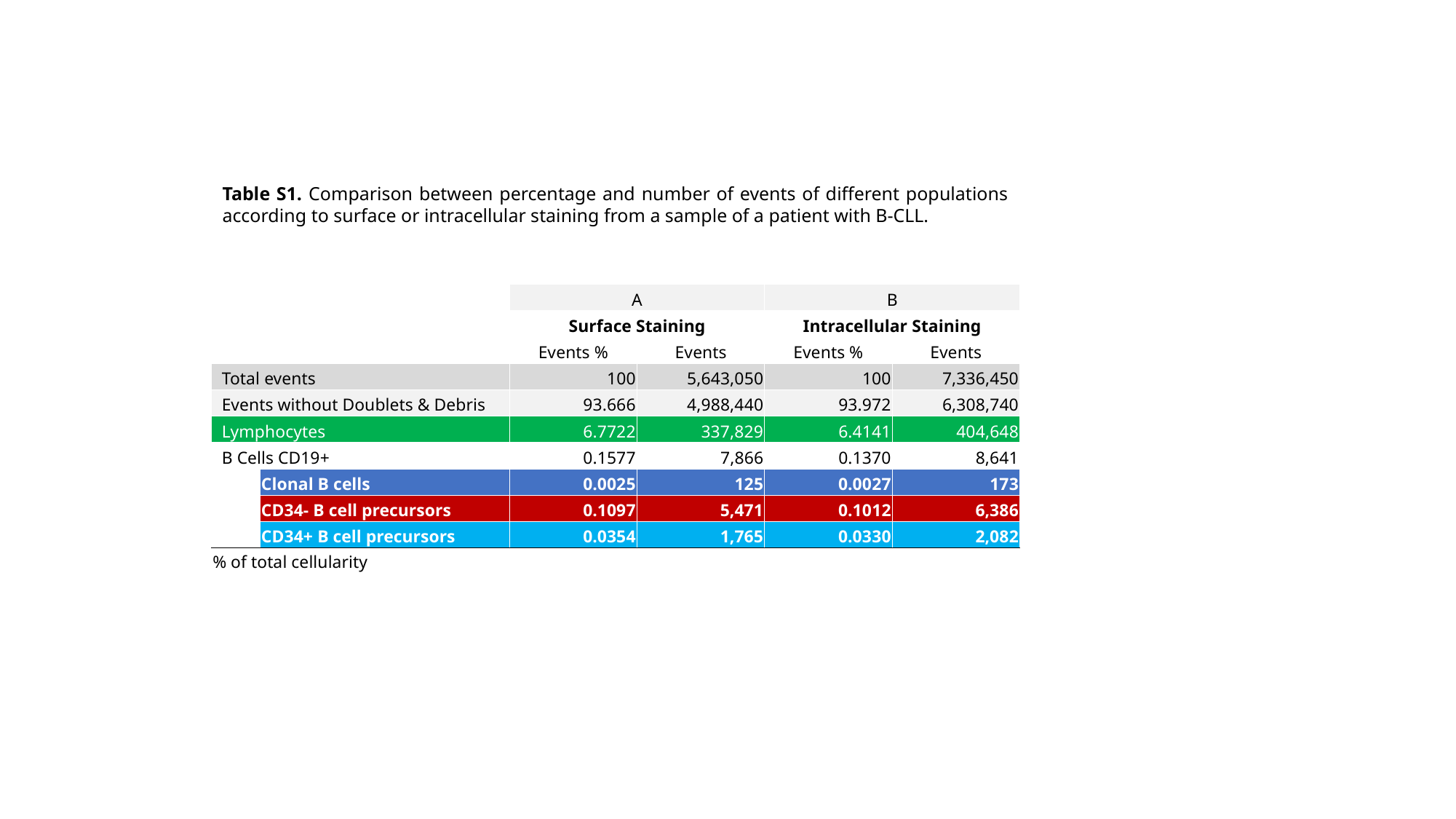

Table S1. Comparison between percentage and number of events of different populations according to surface or intracellular staining from a sample of a patient with B-CLL.
| | | A | | B | |
| --- | --- | --- | --- | --- | --- |
| | | Surface Staining | | Intracellular Staining | |
| | | Events % | Events | Events % | Events |
| Total events | | 100 | 5,643,050 | 100 | 7,336,450 |
| Events without Doublets & Debris | | 93.666 | 4,988,440 | 93.972 | 6,308,740 |
| Lymphocytes | | 6.7722 | 337,829 | 6.4141 | 404,648 |
| B Cells CD19+ | | 0.1577 | 7,866 | 0.1370 | 8,641 |
| | Clonal B cells | 0.0025 | 125 | 0.0027 | 173 |
| | CD34- B cell precursors | 0.1097 | 5,471 | 0.1012 | 6,386 |
| | CD34+ B cell precursors | 0.0354 | 1,765 | 0.0330 | 2,082 |
| % of total cellularity | | | | | |

## Slide 4
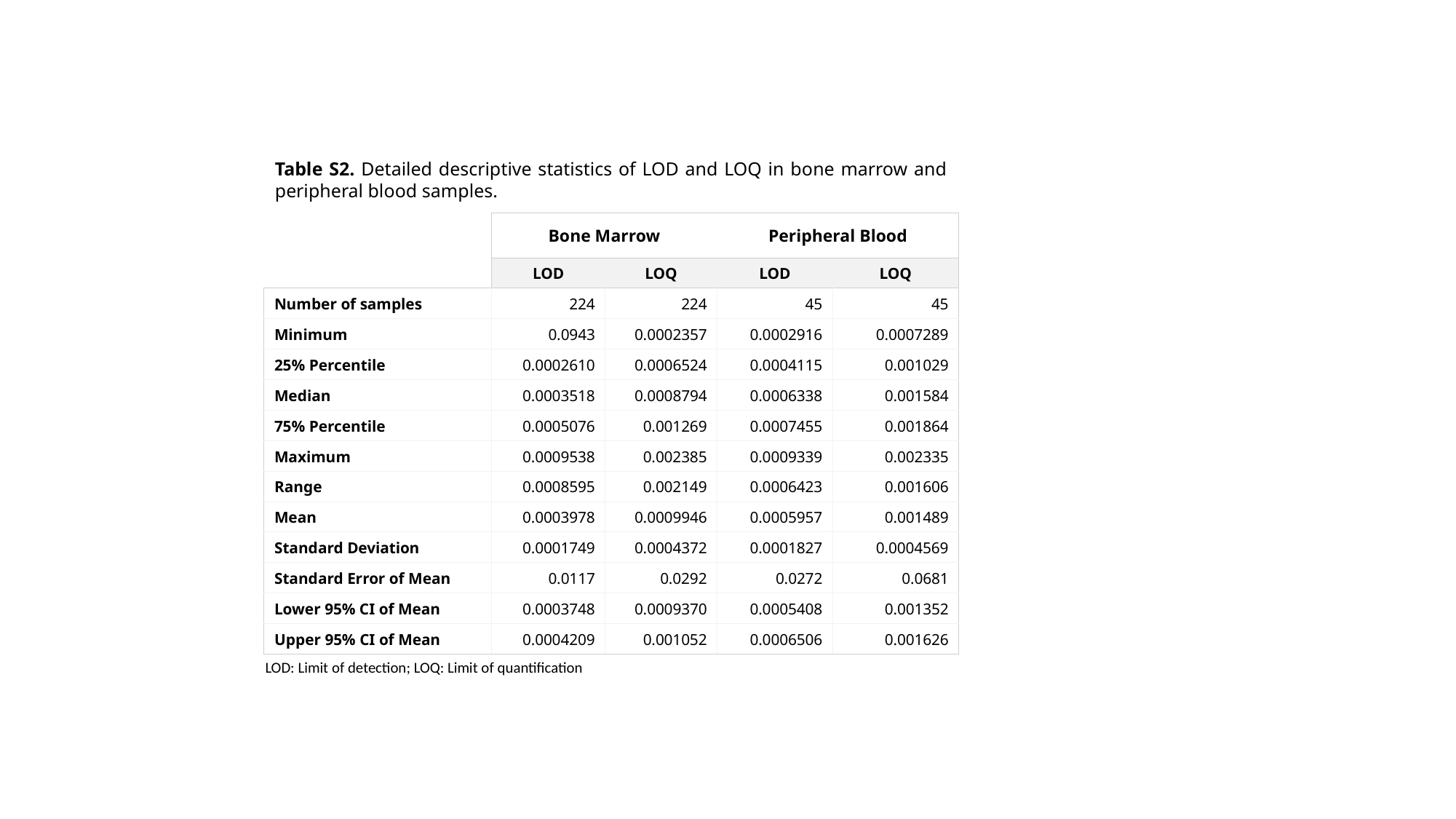

Table S2. Detailed descriptive statistics of LOD and LOQ in bone marrow and peripheral blood samples.
| | Bone Marrow | | Peripheral Blood | |
| --- | --- | --- | --- | --- |
| | LOD | LOQ | LOD | LOQ |
| Number of samples | 224 | 224 | 45 | 45 |
| Minimum | 0.0943 | 0.0002357 | 0.0002916 | 0.0007289 |
| 25% Percentile | 0.0002610 | 0.0006524 | 0.0004115 | 0.001029 |
| Median | 0.0003518 | 0.0008794 | 0.0006338 | 0.001584 |
| 75% Percentile | 0.0005076 | 0.001269 | 0.0007455 | 0.001864 |
| Maximum | 0.0009538 | 0.002385 | 0.0009339 | 0.002335 |
| Range | 0.0008595 | 0.002149 | 0.0006423 | 0.001606 |
| Mean | 0.0003978 | 0.0009946 | 0.0005957 | 0.001489 |
| Standard Deviation | 0.0001749 | 0.0004372 | 0.0001827 | 0.0004569 |
| Standard Error of Mean | 0.0117 | 0.0292 | 0.0272 | 0.0681 |
| Lower 95% CI of Mean | 0.0003748 | 0.0009370 | 0.0005408 | 0.001352 |
| Upper 95% CI of Mean | 0.0004209 | 0.001052 | 0.0006506 | 0.001626 |
| LOD: Limit of detection; LOQ: Limit of quantification | | | | |

## Slide 5
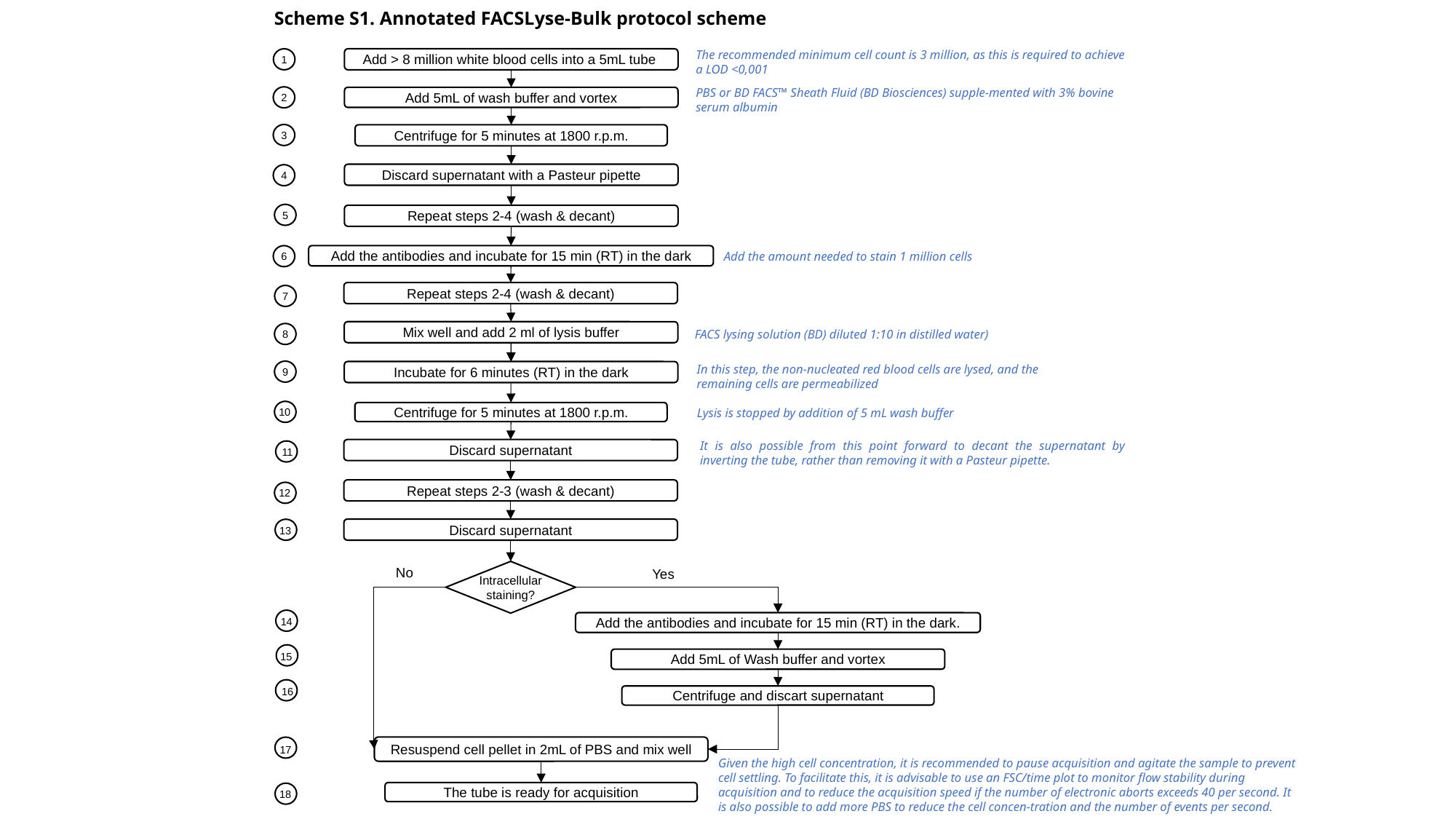

Scheme S1. Annotated FACSLyse-Bulk protocol scheme
The recommended minimum cell count is 3 million, as this is required to achieve a LOD <0,001
1
Add > 8 million white blood cells into a 5mL tube
PBS or BD FACS™ Sheath Fluid (BD Biosciences) supple-mented with 3% bovine serum albumin
2
Add 5mL of wash buffer and vortex
3
Centrifuge for 5 minutes at 1800 r.p.m.
Discard supernatant with a Pasteur pipette
4
5
Repeat steps 2-4 (wash & decant)
Add the amount needed to stain 1 million cells
6
Add the antibodies and incubate for 15 min (RT) in the dark
Repeat steps 2-4 (wash & decant)
7
FACS lysing solution (BD) diluted 1:10 in distilled water)
Mix well and add 2 ml of lysis buffer
8
In this step, the non-nucleated red blood cells are lysed, and the remaining cells are permeabilized
9
Incubate for 6 minutes (RT) in the dark
10
Lysis is stopped by addition of 5 mL wash buffer
Centrifuge for 5 minutes at 1800 r.p.m.
It is also possible from this point forward to decant the supernatant by inverting the tube, rather than removing it with a Pasteur pipette.
Discard supernatant
11
Repeat steps 2-3 (wash & decant)
12
13
Discard supernatant
No
Yes
Intracellular staining?
14
Add the antibodies and incubate for 15 min (RT) in the dark.
15
Add 5mL of Wash buffer and vortex
16
Centrifuge and discart supernatant
Resuspend cell pellet in 2mL of PBS and mix well
17
Given the high cell concentration, it is recommended to pause acquisition and agitate the sample to prevent cell settling. To facilitate this, it is advisable to use an FSC/time plot to monitor flow stability during acquisition and to reduce the acquisition speed if the number of electronic aborts exceeds 40 per second. It is also possible to add more PBS to reduce the cell concen-tration and the number of events per second.
18
The tube is ready for acquisition

## Slide 6
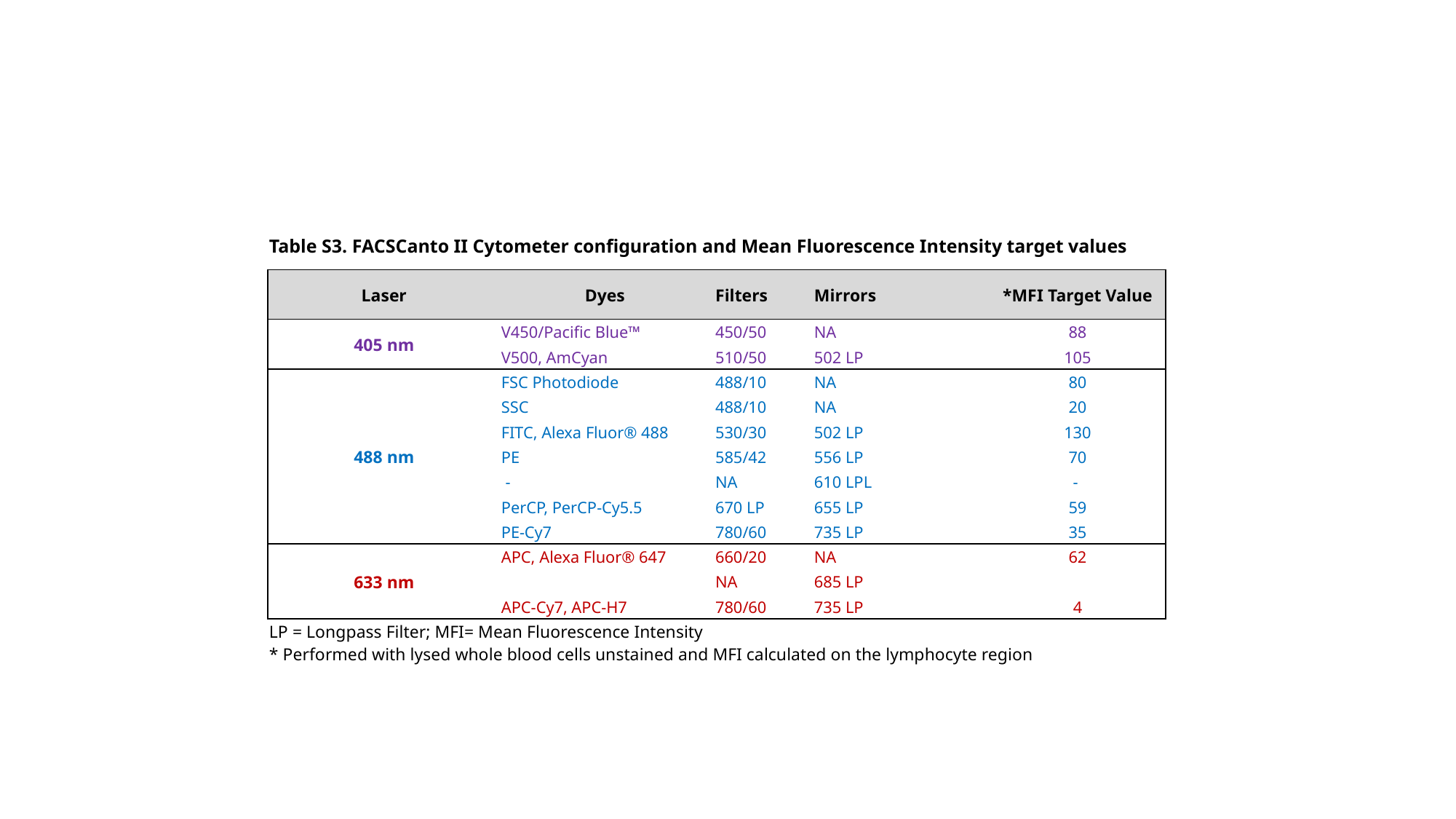

| Table S3. FACSCanto II Cytometer configuration and Mean Fluorescence Intensity target values | | | | |
| --- | --- | --- | --- | --- |
| Laser | Dyes | Filters | Mirrors | \*MFI Target Value |
| 405 nm | V450/Pacific Blue™ | 450/50 | NA | 88 |
| | V500, AmCyan | 510/50 | 502 LP | 105 |
| 488 nm | FSC Photodiode | 488/10 | NA | 80 |
| | SSC | 488/10 | NA | 20 |
| | FITC, Alexa Fluor® 488 | 530/30 | 502 LP | 130 |
| | PE | 585/42 | 556 LP | 70 |
| | - | NA | 610 LPL | - |
| | PerCP, PerCP-Cy5.5 | 670 LP | 655 LP | 59 |
| | PE-Cy7 | 780/60 | 735 LP | 35 |
| 633 nm | APC, Alexa Fluor® 647 | 660/20 | NA | 62 |
| | | NA | 685 LP | |
| | APC-Cy7, APC-H7 | 780/60 | 735 LP | 4 |
| LP = Longpass Filter; MFI= Mean Fluorescence Intensity \* Performed with lysed whole blood cells unstained and MFI calculated on the lymphocyte region | | | | |

## Slide 7
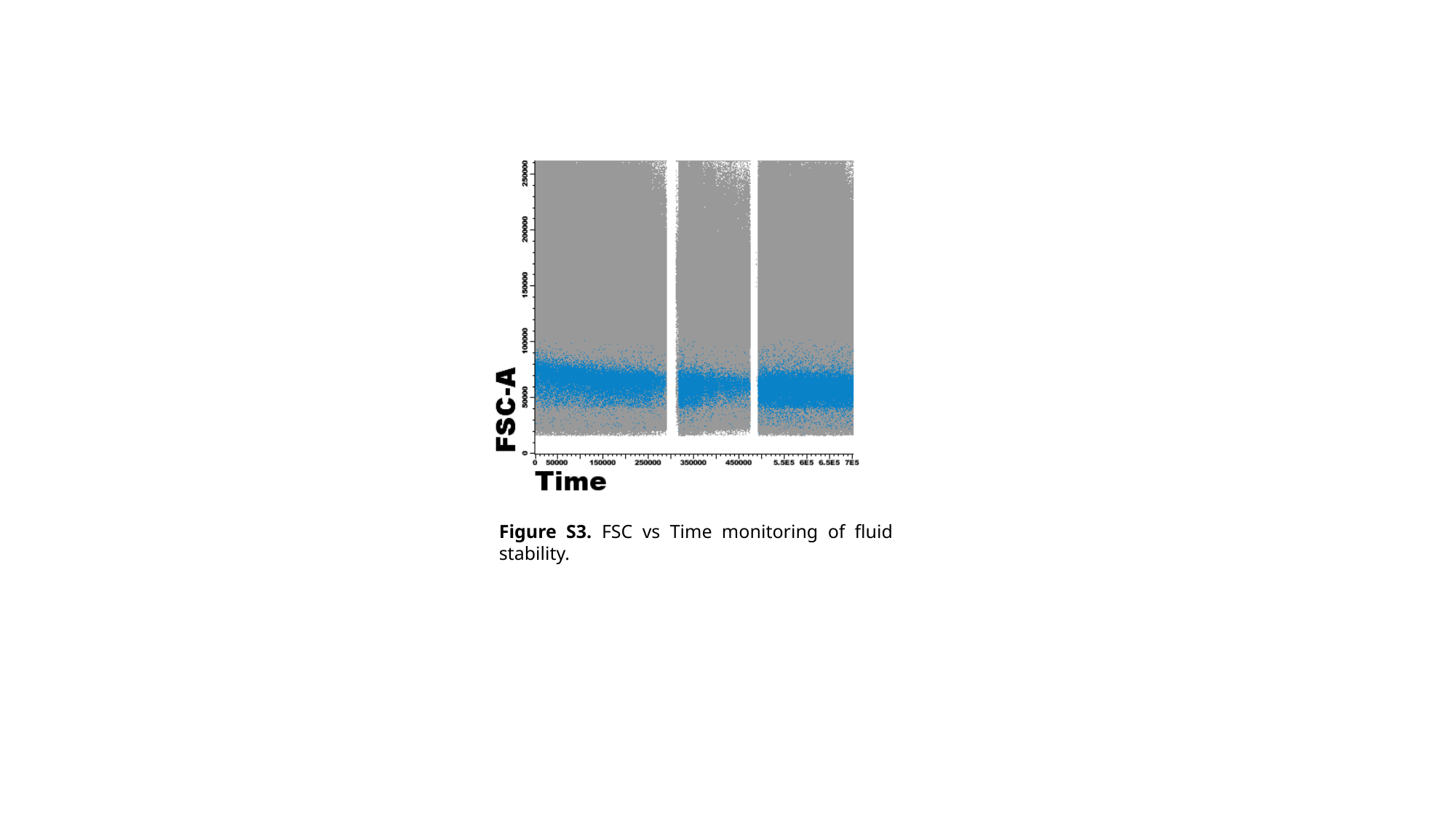

Figure S3. FSC vs Time monitoring of fluid stability.
